# Electrocardiographic changes induced by temperature variations in newly hatched Aperema (*Rhinoclemmys punctularia,* Daudin, 1801)

**DOI:** 10.3389/fvets.2025.1516112

**Published:** 2025-02-11

**Authors:** Deise de Lima Cardoso, Brenda Stefany dos Santos Braga, Daniella Bastos de Araújo, Clarissa Araújo da Paz, Luciana Eiró-Quirino, Thaysa de Sousa Reis, Luana Vasconcelos de Souza, Rayllan da Cunha Ferreira, Gabriela Brito Barbosa, Raíssa Vieira de Souza, Yris da Silva Deiga, Maria Klara Otake Hamoy, Diva Anelie de Araújo Guimarães, Moisés Hamoy

**Affiliations:** ^1^Laboratory of Animal Reproduction, UFPA-ICB, Belém, Brazil; ^2^Post-Graduate Program in Animal Science, UFPA, Belém, Brazil; ^3^Laboratory of Pharmacology and Toxicology of Natural Products, ICB-UFPA, Belém, Brazil

**Keywords:** reptiles, heart rate, temperature, freshwater turtle, Aperema

## Abstract

**Introduction:**

Aperema (*Rhinoclemmys punctularia*) is a South American semi-aquatic freshwater turtle characterized by a highly curved, dark brown to black carapace and is distributed across Central and South America. Climate change affects freshwater turtles in a number of ways, including temperature, hatchling sex, and survival.

**Methods:**

Therefore, we analyzed temperature variations in these turtles through electrocardiographic recordings, since studies on temperature variations in *R. punctularia* are limited.

**Results and discussion:**

Electrocardiography (ECG) is a highly relevant diagnostic tool as it allows for precise assessments of cardiac events and is non-invasive. The development of non-invasive ECG measurement methods is crucial for evaluating and maintaining the health of chelonian individuals during veterinary treatment or experimental procedure. Our findings revealed that heart rate is temperature-dependent, showing that lower environmental temperatures result in decreased heart rates. Therefore, we demonstrated through the electrocardiographic patterns observed during the experiment that a reduction in the ambient temperature to which *Rhinoclemmys punctularia* are exposed can modulate the conductivity and automaticity of cardiac cells, subsequently leading to a decrease in heart rate.

## Graphical abstract

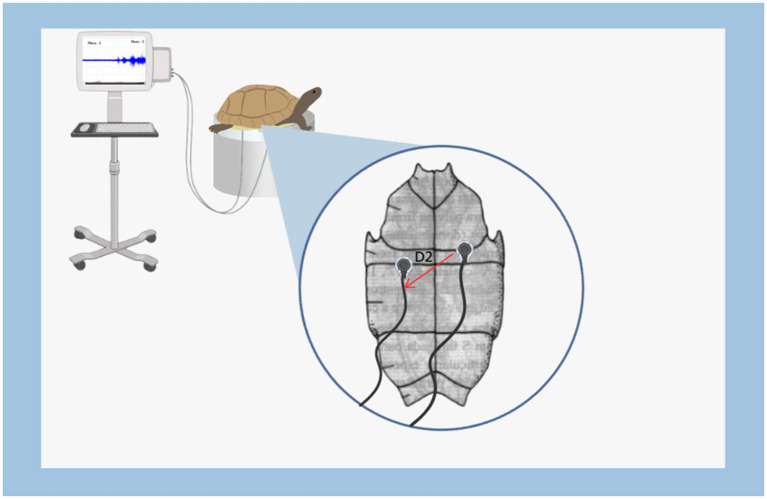

## Introduction

Aperema (*Rhinoclemmys punctularia punctularia*) (Daudin 1801) is a semi-aquatic chelonian widely distributed and commonly referred to as “aperema” in the state of Pará, Brazil. This species is found throughout Central and South America (1–3). It is the only taxon of the Geoemydidae family recorded in Brazil, where it occurs within the Amazon basin, including the states of Amazonas, Amapá, Maranhão, Tocantins, and coastal areas of Pará, with a questionable record for Rio Grande do Norte (1–5).

Relatively few data are available on *R. p. punctularia* (1, 2, 6), and most published studies have focused on the taxonomy, geographic distribution, and captive behavior of this species (1, 4, 7–15).

*Rhinoclemmys punctularia* is a small-sized turtle, averaging 160 mm to 199 mm in length in studies conducted within the Tapajós Basin region, while smaller juveniles measured between 71 mm and 77.8 mm in the Tocantins and Tapajós Basin areas (16). The phalangeal formula 2-3-3-3-2 or 2-3-3-3-3 may be associated with its semi-aquatic behavior, as species with more aquatic lifestyles tend to exhibit three phalanges on the fourth or fifth digit, whereas more terrestrial species typically show a reduction of one phalange on the fourth or fifth digit (17). Based on fecal sample analyses of this species, it was possible to assess its diet, which consists primarily of plant matter, such as seeds from *Myrtaceae* and *Inga* sp., as well as flowers from *Lecythidaceae* (16).

Reptiles are ectothermic animals, as they possess a slow metabolism and rely on external heat sources for body temperature regulation and metabolic rate adjustments (18). Most of the daily activities of species within this class involve interactions between the organism and the external environment, which include adjustments in activity timing and movement from cooler to warmer areas (19). Therefore, temperature is a factor influencing the thermal behavior of these species and, while not the only one, it is the most decisive. This indicates that changes in the local microclimate, particularly environmental alterations near river areas—such as deforestation for agricultural purposes—may impact behavior, essential activities, and possibly the population dynamics of chelonians (20).

Studies have reported a direct and negative correlation between temperature and incubation time, with a statistically significant relationship to the average nest temperature (31.79°C) in newly hatched turtles of the species Hawksbill turtle (*Eretmochelys imbricata*) (21). Under normal conditions, the renal portal system of these animals is active, which facilitates blood flow to the kidneys through valve closure. However, during stress situations, this system becomes inactive, resulting in valve opening and blood redirection to the rest of the circulation. This cardiac shunt complicates respiratory control in these animals (22).

The electrocardiogram is a highly effective non-invasive test for measuring heart rate, analyzing heartbeat rhythm, diagnosing heart irregularities, among other factors (23). In a study of 30 red-eared slider turtles (*Trachemys scripta elegans*), the rhythm was found to be sinus, with sinus arrest occurring in a minority of individuals. Sinus arrest rhythm is more common in ectothermic animals exposed to lower ambient temperatures, resulting in decreased body temperature and reduced basal metabolic rate (24). Therefore, with global warming and constant climate variations, this study aims to contribute to the importance of these temperature variations for the cardiac homeostasis of these animals, which can be altered, compromising the existence and reproduction of these animals.

## Materials and methods

### Animals

In this study, 24 ECG recordings with a duration of 15 min each were utilized. These recordings were taken 24 h after the animals hatching, with an average weight of 32.5 ± 4.5 g, plastron length of 5.5 ± 0.3 cm, plastron width of 4.1 ± 0.3 cm, carapace length of 5.6 ± 0.2 cm, and carapace width of 4.8 ± 0.6 cm. After hatching, the animals were kept at room temperature 25° to 26°. The accommodation was made in a 30x15x10 acrylic box before the experiment exacted. Before the hatching the eggs were kept in incubator. The animals were transferred to the Laboratory of Pharmacology and Toxicology of Natural Products at Federal University of Pará, where they were kept in a temperature-controlled environment. The project was approved by the Federal University of Pará Animal Ethics Committee (CEUA-UFPA) under the number: 4125230223 (ID 002179).

### Electrode fabrication

The electrodes for cardiac signal acquisition were fabricated using 925 silver and soldered onto non-conjugated JST SM cables. They were subsequently insulated with self-polymerizing dental acrylic resin. The non-insulated portion of the electrode was 0.3 mm, designated for cardiac signal acquisition.

### Electrode implantation and physical restraint method

The coordinates for electrocardiogram acquisition were taken from lead D2, with the reference electrode positioned at the fissure between the humeral plates in the median sagittal position. The recording electrode was placed on the abdominal sagittal fissure, forming two points for cardiac vector detection, with each electrode inserted 2.5 mm for ECG recording.

For the physical restraint method, a support was used that suspends the animals, preventing their movement. During restraint, the animals were positioned in ventral decubitus (a normal position for these animals). At the beginning of restraint, the animals attempted to move and displayed signs of agitation, indicative of stress. During this period, electrocardiograms were performed with a duration of 15 min each. All recordings were taken between 8:00 and 11:00 am at the following temperature ranges: A = 25 to 26°C, B = 23 to 24°C, C = 21 to 22°C, and D = 19 to 20°C. Temperature intervals were measured with a Susanda-S brand digital thermometer.

### Electrocardiographic (ECG) signal acquisition

The entire procedure for obtaining the recording was conducted inside a metal-screened Faraday cage. The electrodes were connected to a high-impedance amplifier (Grass Technologies, P511) with a gain of 5,000X, and monitored via an oscilloscope (Protek, 6510). Each recording session lasted 15 min per animal subjected to restraint stress.

### Processing of the obtained data

Offline analysis was conducted using a tool created in Python programming language (version 2.7). The libraries “Numpy” and “Scipy” were used for mathematical processing, and the “Matplotlib” library was employed to generate graphs and plots. A graphical interface was developed using the PyQt4 library. Analyses were performed on the ECG data regarding the power of the recordings, which were examined over the periods of 0–10s and 890–900 s. Morphographic parameters of the electrocardiogram were also analyzed. This morphographic analysis included the following elements of the recordings: heart rate, amplitude of the recording, P-Q and S-T intervals, QRS complex duration, and P wave duration during restraint stress.

### Statistical analysis

Comparisons between the mean amplitude of the tracings and the control values were made using ANOVA followed by Tukey’s *post hoc* test. Mean values are presented with their respective standard deviations (mean ± SD). The significance levels were set as follows: *p < 0.05, **p < 0.01, ***p < 0.001. The software GraphPad® Prism 8 was used for statistical tests and graph creation.

## Results

**Figure 1:**
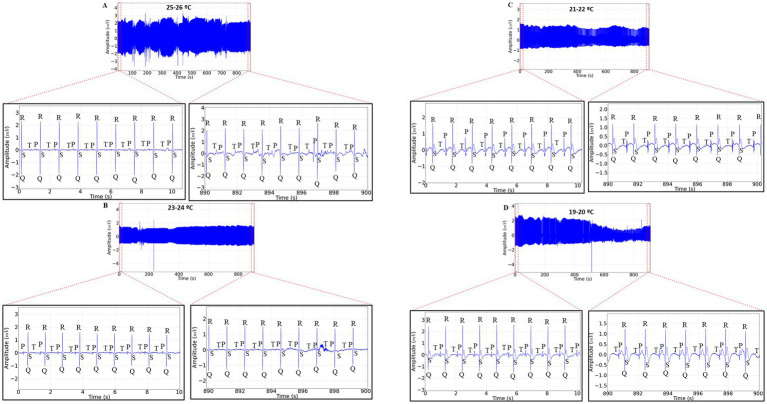
Electrocardiographic (ECG) tracing with a 15-min recording duration in *Rhinoclemmys punctularia*, 24 h post-hatching, showing the analyzed areas of the recordings represented in red dashed lines: 0–10 s (expanded in the lower left) and 890–900 s (expanded in the lower right) in ECGs acquired at the following temperatures: 25 to 26°C **(A)**; 23 to 24°C **(B)**; 21 to 22°C **(C)**; and 19 to 20°C **(D)**.

The 15-min ECG recordings during restraint in newly hatched *Rhinoclemmys punctularia* revealed that at temperature A, heart rate was maintained with similar averages between the beginning and end of the recording (*p* = 0.9944) (Figure 1A). At temperature B, there was also a maintenance of heart rate between the beginning (B1) and the end (B2) of the recording (*p* = 0.999) (Figure 1B). At temperature C, a decrease in heart rate was observed when comparing the start (C1) and the end (C2) of the recording, with a reduction of 28.34% (Figure 1C). At temperature D, there was a 35.98% decrease in amplitude and a 27.49% decrease in heart rate; the beginning of the recording (D1) was higher than the end (D2) (Figure 1D).

**Figure 2:**
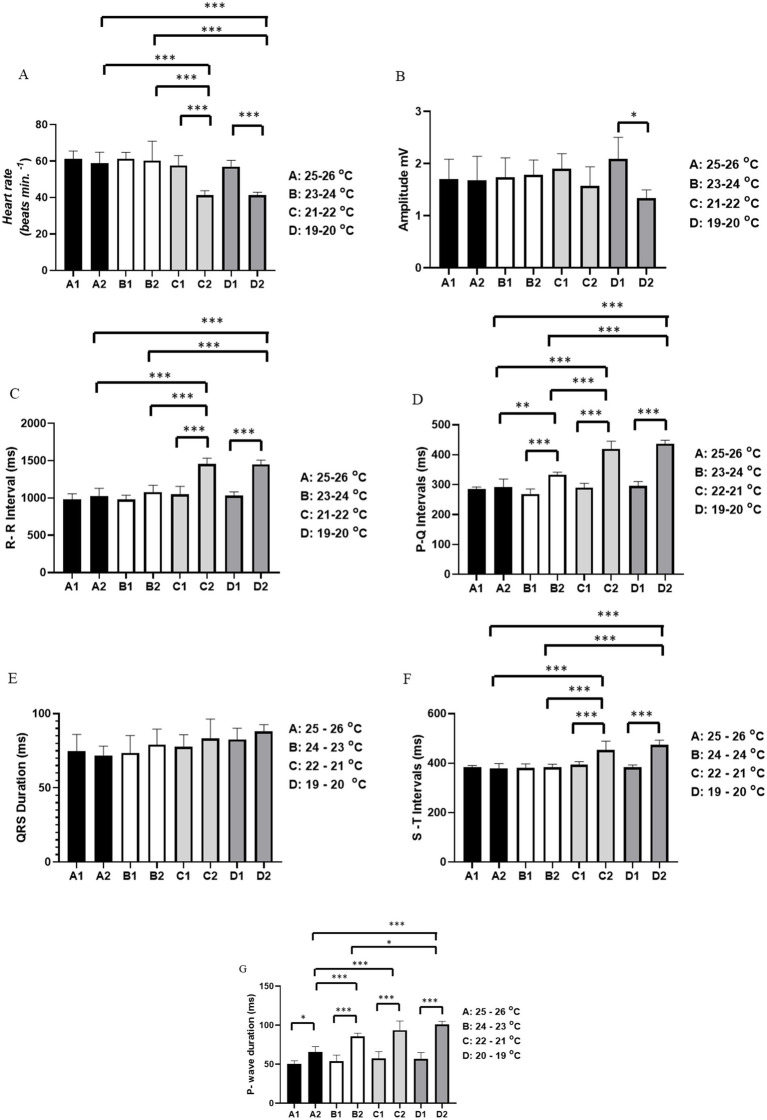
Evaluation of cardiac activity in *Rhinoclemmys punctularia*, 24 h post-hatching, during restraint stress at varying temperatures recorded over 15 min, with assessment conducted between the beginning of the recording (0–10 s) and the end of the recording (890–900 s). The following parameters were evaluated: Heart rate in bpm **(A)**; amplitude in mV **(B)**; R-R interval in ms **(C)**; P-Q interval in ms **(D)**; QRS duration in ms **(E)**; S-T interval in ms **(F)**; and P wave duration in ms **(G)**. Statistical analysis was performed using ANOVA followed by Tukey’s test. *p < 0.05, **p < 0.01, **p < 0.001 (n = 6).

The heart rate at temperature A was initially 61.33 ± 4.32 bpm and at the end of the recording it was 59.00 ± 5.899 bpm (*p* = 0.994). At temperature B, the average heart rate at the start was 61.33 ± 3.502 bpm, similar to the end of the recording at 60.33 ± 10.61 bpm (*p* = 0.999). For temperature C, the average heart rate at the beginning was 57.67 ± 5.42 bpm, which was higher than the heart rate recorded at the end (41.33 ± 2.422 bpm). The average heart rate in period C2 was lower than in periods A2 and B2. At temperature D, the initial heart rate was 57.00 ± 3.521 bpm, which was higher than at the end of the recording (41.33 ± 1.633 bpm). The average heart rate in D2 was lower than in groups A2 and B2 (Figure 2A).

The average amplitude of the QRS complex for temperatures A, B, and C was similar (*p* = 0.5094). However, for the group subjected to temperature D, there was a significant difference between the initial (D1) (2.087 ± 0.419 mV) and final recordings (D2) (1.336 ± 0.1596 mV) (Figure 2B).

For the R-R interval, no differences were observed between groups subjected to temperature A, A1 vs. A2 (*p* = 0.9829), and temperature B, B1 vs. B2 (*p* = 0.4637). The group subjected to temperature C had an average R-R interval at the start of the recording (C1) of 1,048 ± 108.2 ms, which was shorter than at the end (C2) of 1,454 ± 80.15 ms. The period C2 was also longer than periods A2 and B2. The group subjected to temperature D showed an R-R interval in D1 of 1,030 ± 52.81 ms, which was shorter than in D2 (1,450 ± 58.99 ms). The R-R interval during period D2 was longer than periods A2 and B2, but similar to period C2 (*p* = 0.999) (Figure 2C).

For the P-Q interval, animals subjected to temperature A showed similarity between the beginning (A1) and end (A2) of the recordings (*p* = 0.9984). For animals under temperature B, the start of the recording (B1) (268.8 ± 16.75 ms) was shorter than at the end (B2) (333.0 ± 8.922 ms). The P-Q interval for period B2 was longer than period A2. For animals under temperature C, C1 (290.3 ± 14.11 ms) was shorter than C2 (420.0 ± 25.29 ms); likewise, the P-Q interval in C2 was longer than in A2 and B2. For the group subjected to temperature D, the initial recording (295.2 ± 15.65 ms) was shorter than D2 (436.5 ± 11.98 ms). The P-Q intervals for periods D2 and C2 were similar (*p* = 0.719) (Figure 2D).

For the duration of the QRS complex, all groups showed similarity: A1 vs. A2 (*p* = 0.999), B1 vs. B2 (*p* = 0.9683), C1 vs. C2 (*p* = 0.9683), and D1 vs. D2 (*p* = 0.9773) (Figure 2E).

For the S-T interval, animals subjected to temperature A showed similarity between period A1 and period A2 (*p* = 0.9997). At temperature B, periods B1 and B2 were similar (*p* = 0.9999). Animals exposed to temperature C showed a difference between C1 (393.3 ± 25.29 ms), which was shorter than C2 (453.8 ± 35.45 ms). In animals under temperature D, the S-T interval increased during the recording, with D1 (383.5 ± 9.050 ms) being shorter than D2 (473.5 ± 19.32 ms). However, the S-T interval was similar between groups C2 and D2 (*p* = 0.6012) (Figure 2F).

For P wave duration, an increase was observed during the restraint period. In the group exposed to temperature A, A1 (50.67 ± 3.882 ms) was shorter than A2 (65.67 ± 6.947 ms). In animals at temperature B, B1 (53.83 ± 8.035 ms) was shorter than B2 (85.67 ± 4.130 ms), with B2 being longer than A2. For animals under temperature C, the initial part of the recording, C1 (57.33 ± 3.637 ms), was shorter than C2 (93.67 ± 11.79 ms). Animals in C2 had a longer P wave duration compared to groups A2 and B2. In the group at temperature D, D1 (57.00 ± 7.975 ms) had a shorter P wave duration than D2 (101.2 ± 4.07 ms). For P wave duration, D2 was similar to C2 (*p* = 0.6618) (Figure 2G).

## Discussion

This study was the first to describe, using electrocardiography, how the cardiac mechanisms of *Rhinoclemmys punctularia* can be sensitive to temperature variation. We demonstrated that as the ambient temperature decreases, there is a corresponding decrease in heart rate and an increase in the duration of the P wave interval. This indicates a challenge in atrioventricular communication, consistent with the reduction in heart rate.

The evaluation of cardiac function is important for assessing the health status of turtles. Their unique body structure, however, poses challenges, making such assessments difficult to perform. Little attention has been given to the evaluation of cardiac performance and the diagnosis of heart diseases in turtles. The primary challenge in interpreting turtle electrocardiograms stems from the presence of their shell. It is believed that electrical signals may be blocked by hard tissues, such as the carapace, plastron, and bones (25).

The structure of the chelonian heart differs from that of mammals. The turtle heart is a three-chambered structure composed of two atria and a single ventricle, which is partially divided by a muscular ridge (26). The contraction of cardiac muscle in turtles also differs from that in mammals and birds, where electrical activation begins at the sinoatrial node (SA node) and then sequentially propagates to the atrioventricular node (AV node), the bundle of His, and the Purkinje fiber system. A significant difference between mammals and birds lies in the angle of the mean electrical axis (MEA) (27).

There is no SA node, AV node, bundle of His, or Purkinje fiber network forming a specialized conduction system in turtles and terrapins. Instead, the electrical impulse originates from the sinus vein (SV wave), followed by atrial contraction (P wave) and ventricular contraction (QRS complex). SV waves are generally unrecognizable in electrocardiography (ECG) due to their low voltage. After ventricular contraction, the T wave indicates repolarization (28).

In studies conducted by Jackson (29), a proportional relationship was observed between changes in ambient temperature and alterations in heart rate. Jackson explains this relationship in two ways: first, by the reduced affinity between plasma hemoglobin and oxygen molecules at lower temperatures; and second, by the increase in metabolism at higher temperatures. These mechanisms may be associated with the findings of the present study, in which groups C and D, exposed to temperatures of 22–21°C and 20–19°C, respectively, demonstrated a reduction in heart rate in *Rhinoclemmys punctularia*, primarily characterized by an increased RR interval.

The resting metabolic rate of turtles is greatly influenced by temperature and their physiological state. An adequate resting metabolic rate depends on proper tissue perfusion (30). Therefore, cardiac output is closely related to metabolic rate (26, 28). Both heart rate and metabolic rate increase and decrease as the ambient temperature rises and falls, respectively (31).

The QRS complex amplitude decreased in group D, exposed to a temperature of 20–19 degrees Celsius, indicating a reduction in the capacity to conduct electrical impulses between ventricular cardiomyocytes, which ultimately reduces the excitability caused by the generated nodal impulse. According to Mattu et al. (32), as temperature decreases, prolongation of intervals or atrial and ventricular arrhythmias may occur, as well as ECG signs resembling acute myocardial ischemia or myocardial infarction, as observed in the various groups of this study.

With the reduction in temperature, there was also an increase in the duration of the P wave and the PQ interval, indicating a decrease in the conduction capacity from the atrial to the ventricular pump.

Electrocardiography is a valuable diagnostic tool for specific cardiac diseases. However, due to the scarcity of ECG parameter references and standard recording techniques, ECG is rarely performed in turtles and terrapins. Each turtle species has a unique body structure and ECG performance. Several studies have reported ECG performance in specific chelonian species using both invasive and non-invasive methods (33, 34). Invasive methods have included the use of anesthetic drugs and the insertion of probes into the body, which presents a potential risk to the animals’ health. None of these studies achieved ideal ECG recording quality and were also limited by small sample sizes. Therefore, the development of non-invasive ECG measurement methods is crucial for assessing and maintaining the health of chelonian individuals (33, 34).

In conclusion, it was possible to demonstrate, through the electrocardiographic patterns observed during the experiment, that a reduction in the ambient temperature to which *Rhinoclemmys punctularia* are exposed can modulate the conductivity and automaticity of cardiac cells, consequently leading to a decrease in heart rate.

## Data Availability

The original contributions presented in the study are included in the article/supplementary material, further inquiries can be directed to the corresponding author.
